# Hormonal contraceptive use among women living with hypertension in sub-Saharan Africa: insights from 12 countries

**DOI:** 10.1186/s13104-024-06830-8

**Published:** 2024-06-19

**Authors:** Joshua Okyere, Richard Gyan Aboagye, Castro Ayebeng, Abigail Kabukie Dosoo, Samuel Salu, Kwamena Sekyi Dickson

**Affiliations:** 1 Department of Population and Health, University of Cape Coast, Cape Coast, Ghana; 2 Department of Nursing, College of Health Sciences, Kwame Nkrumah University of Science and Technology, Kumasi, Ghana; 3 Department of Family and Community Health, Fred N. Binka School of Public Health, University of Health and Allied Sciences, Hohoe, Ghana; 4 Department of Human Resource Management, School of Business, University of Cape Coast, Cape Coast, Ghana; 5 Department of Epidemiology and Biostatistics, Fred N. Binka School of Public Health, University of Health and Allied Sciences, Ho, Ghana

**Keywords:** Hormonal contraceptive, Hypertension, Women, Sub-Saharan Africa

## Abstract

**Objectives:**

Given the well-established link between hormonal contraceptives and hypertension risk, and the paucity of research on hormonal contraceptive use dynamics in this particular demographic, we hypothesize that there is a likelihood of low utilization of high-risk hormonal contraceptives among women living with hypertension in SSA. This study investigates the prevalence and factors associated with hormonal contraceptive use among women living with hypertension in the SSA.

**Results:**

Only 18.5% of women living with hypertension used hormonal contraceptives. Hormonal contraceptive use was high among women with a higher level of education (aOR = 2.33; 95%CI: 1.73–3.14), those currently working (aOR = 1.38; 95%CI: 1.20–1.59), those who have heard about family planning on the radio (aOR = 1.27, 95%CI: 1.09–1.47), listened to the radio at least once a week (aOR = 1.29, 95%CI: 1.10–1.51), and those residing in rural areas (aOR = 1.32; 95%CI: 1.14–1.54). Conversely, women aged 45–49 exhibited a substantial decrease in the odds of hormonal contraceptive use (aOR = 0.23, 95%CI: 0.14–0.38) compared to younger women (15–19 years). Likewise, the odds of HCU were low among cohabiting (aOR = 0.66; 95%CI: 0.48–0.89) and previously married women (aOR = 0.67; 95%CI: 0.50–0.91) than never married women.

## Background

Contraceptives continue to be a crucial public health intervention worldwide, playing a key role in preventing unintended pregnancies and the associated risks of maternal mortality and morbidity [1, 2]. The utilization of modern contraceptive methods is imperative in fulfilling the sustainable development goals (SDG) target 3.7, which emphasizes the aim for universal availability of sexual and reproductive healthcare services globally for women by 2030 [3, 4]. However, global performance towards achieving the SDG target on family planning and conception stands at 75.7%, with middle and western Africa doing less than 50% [5]. Global unmet need for contraception has declined from 15% in 1990 to 12.6% in 2020, while contraceptive prevalence (modern methods) increased from 19 to 57% during the same period [6]. The proportion of women whose need for family planning is satisfied with modern methods increased from 74% in 2000 to 77% in 2020 [7]. While there has been a global increase in contraception usage, the prevalence rate in sub-Saharan Africa (SSA) remains notably low [8]. The SSA region has the highest unmet need for modern contraception as over 200 million women who do not want to conceive are not using a modern contraceptive method [9, 10]. Evidence from the United Nations Department of Economic and Social Affairs and Population Division showed that 15 SSA countries had more than 20% of the world’s unmet family planning needs [7].

Factors influencing the adoption of hormonal contraceptives vary across countries, regions, and cultures [11]. Numerous studies have delved into the reasons behind the non-usage of hormonal contraceptives, citing concerns like fear of side effects and lack of partner approval, age, marital status, parity, religion, support from parents, contraceptive costs, limited knowledge, peer influence, and misconceptions have shown significant associations with contraceptive use [11–13]. Furthermore, the continuation of hormonal contraceptive methods is linked with the availability of contraceptive methods, easy access to family planning centers, satisfaction with the current method, frequent visits to these centers, attitudes, behavior and skills of health practitioners [14–16]. Women are also less likely to use hormonal contraceptive methods if they have no children, are not told of family planning at a health facility, and have not heard of family planning on the television, radio, or newspaper [17, 18].

Hormonal contraceptives, comprising estrogens and/or progestins, exert cardiovascular effects, impacting blood pressure [19]. The prevalence of oral contraceptive (OC) use globally stands at 151 million users, making it the most commonly used hormonal contraceptive method [7]. Despite reduced OC doses, epidemiological evidence maintains a smaller yet noteworthy association between OC pills and hypertension [20, 21]. Combined oral contraceptives (COC) are associated with hypertension in about 2% of women, contributing to an average systolic blood pressure increase of 7–8 mmHg [22]. Afshari et al. [23] reported a 25% hypertension prevalence among women using OCs, with a 1.23 to 1.96 times higher risk compared to non-users. Women between the ages of 15 and 45 who used hormonal contraceptives within 30 days of a hypertension diagnosis exhibited a significantly elevated risk of developing hypertension [24]. Additionally, prolonged OC use during fertile years could also emerge as a notable risk factor for subsequent hypertension post-menopause [25, 26].

Despite extensive research on hormonal contraceptive use in the general population within SSA [12, 27–29], there is a notable scarcity of information concerning hormonal contraceptive use among women with hypertension in the sub-Saharan Africa (SSA) region. Given the well-established link between hormonal contraceptives and hypertension risk, and the paucity of research on hormonal contraceptive use dynamics in this particular demographic, we hypothesize that there is a likelihood of low utilization of high-risk hormonal contraceptives among women living with hypertension in SSA. Consequently, our study aims to bridge this knowledge gap by investigating the prevalence of and factors associated with hormonal contraceptive use among women living with hypertension in the SSA.

## Methods

### Data source and design

Data used for this study was from the Demographic and Health Survey (DHS) of twelve (12) countries in SSA. We used the most current DHS data of the respective countries (see Table 1). Specifically, we pooled data from the individual recode files of the 12 countries: Benin, Burundi, Cote d’Ivoire, Cameroon, Gabon, Gambia, Kenya, Madagascar, Mali, Mauritania, Tanzania, and Zambia. These countries were selected because they have the most recent dataset (2017–2022), and also because it contained an indicator for measuring hypertension. DHS is a comprehensive survey conducted in more than 85 low- and middle-income countries globally. The survey adopts a descriptive cross-sectional design, employing a two-stage cluster sampling method to select respondents. The detailed sampling technique has been thoroughly outlined in the literature [30]. The data collection process utilizes standardized structured questionnaires to gather information from respondents on various health and social indicators, encompassing aspects such as risky sexual behavior. In this study, we included a total sample of 7,324 women living with hypertension (see Table 1).

### Measures

#### Outcome variable

Hormonal contraceptive use constituted the outcome variable for this study. Participants were requested to specify their current contraceptive method. Instances, where women reported using an intrauterine device (IUD) and other modern methods, were excluded due to insufficient details regarding the specific type of IUD and modern methods, respectively. Women who identified using oral contraceptives, injectable, and implants were categorized as users of hormonal contraceptives, denoted by the code “1 = yes.” Conversely, women not utilizing any hormonal contraceptives were coded as “0 = no.”

### Explanatory variables

Informed by extant literature [11–13], as well as the availability of variables in the dataset, 15 explanatory variables were considered. These include the age of the woman, educational level, marital status, current working status, number of children ever born, having visited the health facility in the last 12 months, having heard of family planning on radio, television or in the magazines or newspapers, frequency of watching television, listening to the radio or reading newspapers or magazines, internet usage, wealth index, and place of residence. We further grouped the variables into individual and contextual level. Except for household wealth index and place of residence which were contextual level variables, the remaining variables were placed at the individual level.

### Statistical analyses

The analyses were performed in STATA version 17. First, we used percentages with confidence intervals to summarize the proportion of women living with hypertension who used hormonal contraceptives. Next, the Pearson chi-square test of independence was adopted to examine the distribution of hormonal contraceptive use among hypertensive women across the explanatory variables as well as determine the association between these variables. We included only variables with p-values less than or equal to 0.05 in the regression analysis. Afterwards, a multilevel binary logistic regression analysis was used to examine the factors associated with hormonal contraceptive use among women living with hypertension. To ascertain these factors, four models were used. The first model was an empty one that measured the variation in hormonal contraceptive attributed to the primary sampling unit (PSU). We placed the individual level and contextual-level variables in Model II and III, respectively. The fourth model (Model IV) contained all the explanatory variables. The results were presented in two forms: fixed effect and random effect. The fixed effect results showed the association between the explanatory variables and hormonal contraceptive use. The results were presented using adjusted odds ratio (aOR) with their 95% CIs. On the other hand, the random effect results measure the fitness of the models as well as ensuring model comparability measured using the Akaike’s Information Criterion (AIC) and log likelihood values, where the model with the least AIC and highest log likelihood values were selected as the best-fitted model from where the results were described and discussed. All analyses were weighted, as well as, using the STATA command “svyset” which takes into account the complex design of the survey.

### Ethical considerations

Our study did not require ethical clearance as the dataset is publicly available. However, we obtained permission from the Monitoring and Evaluation to Assess and Use Results Demographic and Health Surveys (MEASURE DHS) before utilizing the dataset.

## Results

### Prevalence of hormonal contraceptive use among women living with hypertension

Overall, 18.5% of women living with hypertension used hormonal contraceptives. Madagascar reported the largest proportional (34.1%) use of hormonal contraceptives among this cohort while Gabon reported the lowest contraceptive use (5.0%) (see Table 1).

**Table 1:** Sample of women included in the study and prevalence of hormonal contraceptives

Country	Survey year	Unweighted sample	Weighted sample	Hormonal contraceptive use
Benin	2017-18	527	591	8.9 [6.7, 11.7]
Burundi	2016-17	142	536	7.8 [4.2, 13.9]
Cote d’Ivoire	2021	396	615	13.8 [9.8, 19.1]
Cameroon	2018	671	527	8.5 [6.5, 11.1]
Gabon	2019–2021	619	491	5.0 [3.1, 8.2]
Gambia	2019–2020	555	471	16.5 [13.5, 20.7]
Kenya	2022	974	1,319	30.7 [27.1, 34.5]
Madagascar	2021	763	738	34.1 [30.5, 38.0]
Mali	2018	633	392	14.0 [10.9, 17.9]
Mauritania	2019–2021	427	594	11.0 [7.8, 15.3]
Tanzania	2022	755	594	19.3 [15.8, 23.2]
Zambia	2018	755	549	28.7 [25.0, 32.6]
**Total**		**7,324**	**7,324**	**18.5 [17.3, 19.8]**

### Distribution of hormonal contraceptive use by explanatory variables

The results in Table 2 showcase the relationship between various background characteristics of the respondents and hormonal contraceptive use. Notably, statistically significant variations were observed across different age groups. Among women aged 30–34, the highest proportion of hormonal contraceptive use was reported at 25.3% (CI: 21.8, 29.1). Additionally, higher hormonal contraceptive use was found among those with a primary education [24.4% (CI: 22.1, 26.9)], married women [21.6% (CI: 20.0, 23.3)], those who were employed [20.2% (CI: 18.7, 21.7)], and those with a parity of three [23.7% (CI: 20.6, 27.0)].

The prevalence of hormonal contraceptive use was also high among those who had heard about family planning on radio (22.5%, CI: 20.6, 24.5) and television (22.2%, CI: 20.2, 24.5) (*p* < 0.001). The frequency of listening to the radio also revealed a significant impact, with the highest proportion observed among those who listened at least once a week (23.0%, CI: 21.1, 25.1) (*p* < 0.001). Also, hormonal contraceptive use prevalence was higher among women living with hypertension who were in the richer quintile [21.6% (CI: 19.0, 24.5)] and rural residents [20.5% (CI: 18.7, 22.4)].

**Table 2:** Distribution of hormonal contraceptive use by explanatory variables

Variables	Weighted sample	Hormonal contraceptive use	
Women’s age (years)			
15–19	425 (5.8)	6.4 [4.0, 10.0]	< 0.001
20–24	766 (10.5)	16.4 [13.0, 20.5]	
25–29	1006 (13.7)	23.5 [20.0, 27.5]	
30–34	1084 (14.8)	25.3 [21.8, 29.1]	
35–39	1242 (17.0)	23.8 [20.7, 27.1]	
40–44	1409 (19.2)	17.3 [14.8, 20.2]	
45–49	1392 (19.0)	11.1 [9.2, 13.3]	
**Level of education**			< 0.001
No education	1,768 (24.1)	10.7 [8.9, 12.9]	
Primary	2,357 (32.2)	24.4 [22.1, 26.9]	
Secondary	2,490 (34.0)	18.6 [16.6, 20.8]	
Higher	709 (9.7)	18.0 [14.4, 22.2]	
**Marital status**			< 0.001
Never married	1,065 (14.6)	11.5 [8.9, 14.7]	
Married	4,191 (57.2)	21.6 [20.0, 23.3]	
Cohabiting	938 (12.8)	18.0 [14.6, 22.0]	
Previously married	1,130 (15.4)	14.2 [11.7, 17.1]	
**Current working status**			< 0.001
Not working	2,326 (31.8)	15.0 [13.1, 17.2]	
Working	4,998 (68.2)	20.2 [18.7, 21.7]	
**Number of children ever born**			< 0.001
Zero	981 (13.4)	4.6 [2.7, 7.7]	
One	1007 (13.8)	17.1 [14.1, 20.6]	
Two	1125 (15.3)	23.7 [20.5, 27.2]	
Three	1215 (16.6)	23.7 [20.6, 27.0]	
Four or more	2996 (40.9)	19.5 [17.8, 21.4]	
**Visited health facility in last 12 months**			0.557
No	2,723 (37.2)	18.0 [16.1, 20.2]	
Yes	4,601 (62.8)	18.8 [17.3, 20.4]	
**Heard family planning on television last few months**			< 0.001
No	4,777 (65.2)	16.5 [15.1, 18.1]	
Yes	2,547 (34.8)	22.2 [20.2, 24.5]	
**Heard family planning on radio last few months**			< 0.001
No	4,183 (57.1)	15.5 [14.0, 17.2]	
Yes	3,141 (42.9)	22.5 [20.6, 24.5]	
**Heard family planning in newspaper/magazine last few months**			0.220
No	6,361 (86.9)	18.2 [17.0, 19.6]	
Yes	963 (13.1)	20.5 [17.3, 24.1]	
**Frequency of watching television**			0.598
Not at all	2,577 (35.2)	18.6 [16.6, 20.8]	
Less than once a week	1,158 (15.8)	17.1 [14.5, 20.1]	
At least once a week	3,589 (49.0)	18.9 [17.2, 20.7]	
**Frequency of listening to radio**			< 0.001
Not at all	2,545 (34.8)	13.7 [12.1, 15.5]	
Less than once a week	1,644 (22.4)	17.4 [15.0, 20.1]	
At least once a week	3,135 (42.8)	23.0 [21.1, 25.1]	
**Frequency of reading newspaper or magazine**			0.265
Not at all	5,729 (78.2)	18.0 [16.7, 19.4]	
Less than once a week	947 (12.9)	20.6 [17.3, 24.3]	
At least once a week	648 (8.9)	20.3 [16.3, 25.0]	
**Internet usage**			0.278
No	4,558 (62.2)	19.1 [17.6, 20.6]	
Yes	2,766 (37.8)	17.6 [15.6, 19.8]	
**Wealth index**			0.002
Poorest	659 (9.0)	16.8 [13.7, 20.3]	
Poorer	1,008 (13.8)	15.3 [12.9, 18.1]	
Middle	1,312 (17.9)	20.6 [18.0, 23.4]	
Richer	1,767 (24.1)	21.6 [19.0, 24.5]	
Richest	2,578 (35.2)	17.1 [15.1, 19.2]	
**Place of residence**			0.004
Urban	4,018 (54.9)	16.9 [15.3, 18.6]	
Rural	3,306 (45.1)	20.5 [18.7, 22.4]	

*P-values obtained from chi-square test

### Factors associated with hormonal contraceptive use among the women with hypertension

Women aged 45–49 exhibited a substantial decrease in the odds of hormonal contraceptive use (aOR = 0.23, 95%CI: 0.14–0.38) compared to younger women (15–19 years). Compared to women with no formal education, those with a higher level of education (aOR = 2.33; 95%CI: 1.73–3.14) reported higher odds of using hormonal contraceptives. Marital status also played a role, with cohabiting (aOR = 0.66; 95%CI: 0.48–0.89) and previously married women (aOR = 0.67; 95%CI: 0.50–0.91) showing lower odds compared to never married women. The number of children ever born demonstrated a strong positive association with hormonal contraceptive use, with increasing odds for higher numbers of children (*p* < 0.001). Working women had higher odds of hormonal contraceptive use (aOR = 1.38; 95%CI: 1.20–1.59) compared to those not working (see Table 3).

Women who reported hearing about family planning on the radio exhibited increased odds of hormonal contraceptive use (aOR = 1.27, 95%CI: 1.09–1.47). Also, women who listened to the radio at least once a week had higher odds of hormonal contraceptive use (aOR = 1.29, 95%CI: 1.10–1.51) compared to those who did not listen at all. Additionally, residing in a rural area was associated with increased odds of hormonal contraceptive use (aOR = 1.32; 95%CI: 1.14–1.54).

**Table 3:** Factors associated with hormonal contraceptive use among women living with hypertension

Variable	Model I Empty model	Model II aOR [95% CI]	Model III aOR [95% CI]	Model IV aOR [95% CI]
Fixed effect results				
Women’s age (years)				
15–19		1.00		1.00
20–24		1.04 [0.65, 1.65]		1.06 [0.66, 1.68]
25–29		0.96 [0.60, 1.53]		1.00 [0.62, 1.60]
30–34		0.81 [0.50, 1.30]		0.86 [0.53, 1.39]
35–39		0.64 [0.40, 1.04]		0.69 [0.42, 1.12]
40–44		0.44^***^ [0.27, 0.72]		0.48^**^ [0.29, 0.78]
45–49		0.22^***^ [0.13, 0.36]		0.23^***^ [0.14, 0.38]
**Level of education**				
No education		1.00		1.00
Primary		2.78^***^ [2.31, 3.33]		2.80^***^ [2.33, 3.36]
Secondary		2.10^***^ [1.73, 2.56]		2.23^***^ [1.83, 2.73]
Higher		2.09^***^ [1.56, 2.79]		2.33^***^ [1.73, 3.14]
**Marital status**				
Never married		1.00		1.00
Married		1.03 [0.80, 1.34]		1.00 [0.77, 1.29]
Cohabiting		0.68^*^ [0.50, 0.92]		0.66^**^ [0.48, 0.89]
Previously married		0.70^*^ [0.52, 0.94]		0.67^**^ [0.50, 0.91]
**Number of children ever born**				
Zero		1.00		1.00
One		5.79^***^ [3.81, 8.79]		5.76^***^ [3.78, 8.76]
Two		9.40^***^ [6.08, 14.55]		9.41^***^ [6.07, 14.60]
Three		12.12^***^ [7.77, 18.92]		11.99^***^ [7.66, 18.76]
Four or more		14.87^***^ [9.51, 23.26]		14.44^***^ [9.20, 22.68]
**Current working status**				
Not working		1.00		1.00
Working		1.40^***^ [1.21, 1.61]		1.38^***^ [1.20, 1.59]
**Heard family planning on television last few months**				
No		1.00		1.00
Yes		1.03 [0.88, 1.19]		1.09 [0.93, 1.28]
**Heard family planning on radio last few months**				
No		1.00		1.00
Yes		1.33^***^ [1.14, 1.54]		1.27^**^ [1.09, 1.47]
**Frequency of listening to radio**				
Not at all		1.00		1.00
Less than once a week		0.98 [0.82, 1.18]		1.00 [0.83, 1.19]
At least once a week		1.28^**^ [1.10, 1.50]		1.29^**^ [1.10, 1.51]
**Wealth index**				
Poorest			1.00	1.00
Poorer			0.94 [0.74, 1.20]	0.81 [0.63, 1.05]
Middle			1.41^**^ [1.12, 1.76]	1.18 [0.93, 1.49]
Richer			1.54^***^ [1.23, 1.93]	1.17 [0.92, 1.49]
Richest			1.24 [0.98, 1.56]	0.94 [0.72, 1.22]
**Place of residence**				
Urban			1.00	1.00
Rural			1.45^***^ [1.25, 1.67]	1.32^***^ [1.14, 1.54]
**Random effect model**				
PSU variance (95% CI)	0.141 [0.070, 0.286]	0.078 [0.025, 0.250]	0.143 [0.071, 0.288]	0.074 [0.022, 0.249]
ICC	0.041	0.023	0.042	0.022
Wald chi-square	Reference	507.23***	52.14***	533.88***
**Model fitness**				
Log-likelihood	-3532.8168	-3199.5995	-3506.687	-3182.3081
AIC	7069.634	6445.199	7027.374	6420.616
N	7324	7324	7324	7324
Number of clusters	1,088	1,088	1,088	1,088

aOR = adjusted odds ratios; CI = Confidence Interval; ^*^p < 0.05, ^**^p < 0.01, ^***^p < 0.001; 1.00 = Reference category; PSU = Primary Sampling Unit; ICC = Intra-Class Correlation; AIC = Akaike’s Information Criterion

## Discussion

This study aimed to examine the prevalence and factors associated with the use of hormonal contraceptives among women living with hypertension in SSA. Our study revealed that less than a quarter (18.5%) of women living with hypertension utilized hormonal contraceptives. The observed low utilization of hormonal contraceptives among this cohort is not surprising as previous studies [22–26, 31] have found a higher risk of high blood pressure among those who use this type of contraceptive. As such, the low utilization of hormonal contraceptives may be a reflection of participants’ awareness of the risk that this type of contraceptive poses to their blood pressure. Notwithstanding, we found that hormonal contraceptive utilization among women living with hypertension was influenced by age, marital status, employment status, access to health information and messages, place of residence, and parity.

Regarding age, we found that older women of reproductive age living with hypertension were significantly less likely to use hormonal contraceptives than younger women. It is plausible that older women may be more likely to be aware of their hypertension status [32]; hence, influencing them to opt for other forms of contraception than hormonal contraceptive use. Moreover, healthcare providers may be more conservative in prescribing hormonal contraceptives to older individuals with hypertension, emphasizing alternative contraceptive methods or highlighting potential health risks.

Hypertensive women with higher educational attainment were 2.3 times more likely to use hormonal contraceptives than those with no formal education. The result is consistent with Toffol et al.’s study [33] which found a positive association between educational level and hormonal contraceptive use. Similar findings have been reported in Ethiopia [34]. One plausible explanation is that women with higher educational levels may have better access to information and healthcare resources, enabling them to make informed decisions about family planning. Additionally, women with higher educational attainment may have greater financial independence, allowing them to afford and access a wider range of contraceptive options.

Our study revealed that exposure to the radio and exposure to family planning messages on the radio increased the odds of hormonal contraceptive use among women living with hypertension. This aligns with Fenta and Gebremichael’s study [24] which reported a significant positive association between media exposure and hormonal contraceptive use. In many SSA countries, hormonal contraceptives – particularly oral pills – are widely advertised by manufacturers and health advocates on radio [35, 36]. It is, therefore, possible that women living with hypertension would be more exposed to information about hormonal contraceptives, and barrier methods like condoms than any other type of contraceptives.

Consistent with findings from Bangladesh [37], we found that currently employed women living with hypertension have a 1.38 times higher likelihood of using hormonal contraceptives than those who were not working. Being employed may signify some level of stability and autonomy that enables women to make proactive decisions about their reproductive health. Furthermore, employed women may also have greater financial resources, which can facilitate access to hormonal contraceptives that may otherwise be financially burdensome. It is also possible that the workplace environment may promote discussions about family planning and reproductive health, leading to increased awareness and uptake of hormonal contraceptives among employed women living with hypertension.

Surprisingly, the findings indicate that rural residency is associated with 32% higher likelihood of using hormonal contraceptives. This is inconsistent with a previous study [37] that found lower hormonal contraceptive use among rural dwelling women. Nonetheless, the observed association aligns with Skiba et al. [38] who found a significant positive association between rural residency and hormonal contraceptive use. This is a novel finding; perhaps it could be explained from the perspective that family planning advocacy in SSA has focused much attention on rural areas. Previous studies have found hormonal contraceptives to be associated with higher hypertension risk [22–24]. Therefore, it is plausible that urban women possess greater access to comprehensive safety information regarding the risk that hormonal contraceptives pose, hence, contributing to reduced usage rates. Further research is required to fully understand this pattern of association. We also found high odds of hormonal contraceptive use among parous women – a result that is corroborated by prior evidence from Australia [38].

### Strengths and limitations

This research addresses a significant knowledge gap in SSA in terms of hormonal contraceptives. Also, our study provided a detailed description and applied appropriate statistical analyses; thus, guaranteeing the validity and replicability of our study to similar contexts. The large sample size used helps us to generalize the findings to the wider population of SSA women living with hypertension. Yet, there are some important limitations. Causality or temporal relationships cannot be established due to the cross-sectional nature of the demographic and health survey. Additionally, it is worth noting that there is a possibility of recall bias due to the reliance on self-reported data on contraceptive use. Our analysis excluded women who used IUD, as such, there is a possibility of an underestimation in the reported prevalence of hormonal contraceptive use. Another important limitation is the fact that the DHS does not provide data on the dosage and type of hormonal contraceptive used. Also, other key confounders such as cultural beliefs and self-efficacy were not assessed due to their absence in the DHS. As we relied on a secondary dataset, we were unable to account for the actual family planning messages and content conveyed through the various media platforms. Hence, caution should be exercised when interpreting the findings.

## Conclusion

Our study highlights the low utilization rate of hormonal contraceptives among women living with hypertension in SSA. We conclude that increasing age, higher educational attainment, rural residency, exposure to radio, and exposure to family planning messages are key factors that promote the use of hormonal contraceptives among this demographic. As such, tailored interventions are warranted to address barriers to access and increase awareness of contraceptive options among this vulnerable population. Additionally, healthcare providers should engage in informed discussions with patients, considering individual health profiles and preferences to promote safe and effective contraceptive choices. Further research is recommended to explore the observed rural-urban disparity in hormonal contraceptive use among hypertensive women in SSA and to inform targeted interventions effectively. Additionally, future studies should consider assessing the specific types of hormonal contraceptives, as well as changes in contraceptive methods used by women living with hypertension before, and after diagnosis of their condition.

## Data Availability

The datasets generated and/or analysed during the current study are available in the Measure DHS repository: https://www.dhsprogram.com/data/available-datasets.cfm.

## Abbreviations

aOR: Adjusted Odds Ratio
CI: Confidence Interval
COC: Combined Oral Contraceptives
OC: Oral Contraceptives
